# Induction of labour care in the UK: A cross-sectional survey of maternity units

**DOI:** 10.1371/journal.pone.0297857

**Published:** 2024-02-28

**Authors:** Beck Taylor, Fiona Cross-Sudworth, Michael Rimmer, Laura Quinn, R. Katie Morris, Tracey Johnston, Sharon Morad, Louisa Davidson, Sara Kenyon, UK Audit and Research Collaborative in Obstetrics and Gynaecology (UKARCOG) Members

**Affiliations:** 1 Warwick Medical School, University of Warwick, Coventry, United Kingdom; 2 Institute of Applied Health Research, University of Birmingham, Birmingham, United Kingdom; 3 Medical Research Council Centre for Reproductive Health, The Queen’s Medical Research Institute, University of Edinburgh, Edinburgh, United Kingdom; 4 Professor of Obstetrics and Maternal Fetal Medicine, Institute of Applied Health Research, University of Birmingham, Birmingham, United Kingdom; 5 Birmingham Women’s and Children’s NHS Foundation Trust, Birmingham, United Kingdom; 6 Professor of Evidence Based Maternity Care, Institute of Applied Health Research, University of Birmingham, Birmingham, United Kingdom; Werabe University, ETHIOPIA

## Abstract

**Objectives:**

To explore local induction of labour pathways in the UK National Health Service to provide insight into current practice.

**Design:**

National survey.

**Setting:**

Hospital maternity services in all four nations of the UK.

**Sample:**

Convenience sample of 71 UK maternity units.

**Methods:**

An online cross-sectional survey was disseminated and completed via a national network of obstetrics and gynaecology specialist trainees (October 2021-March 2022). Results were analysed descriptively, with associations explored using Fisher’s Exact and ANOVA.

**Main outcome measures:**

Induction rates, criteria, processes, delays, incidents, safety concerns.

**Results:**

54/71 units responded (76%, 35% of UK units). Induction rate range 19.2%-53.4%, median 36.3%. 72% (39/54) had agreed induction criteria: these varied widely and were not all in national guidance. Multidisciplinary booking decision-making was not reported by 38/54 (70%). Delays reported ‘often/always’ in hospital admission for induction (19%, 10/54) and Delivery Suite transfer once induction in progress (63%, 34/54). Staffing was frequently reported cause of delay (76%, 41/54 ‘often/always’). Delays triggered incident reports in 36/54 (67%) and resulted in harm in 3/54 (6%). Induction was an area of concern (44%, 24/54); 61% (33/54) reported induction-focused quality improvement work.

**Conclusions:**

There is substantial variation in induction rates, processes and policies across UK maternity services. Delays appear to be common and are a cause of safety concerns. With induction rates likely to increase, improved guidance and pathways are critically needed to improve safety and experience of care.

## Introduction

Induction of labour (IOL) rates are increasing in the UK, and in 2019 a third of women were induced, with the rate for nulliparous women as high as 36% [1]. Rates are increasing worldwide, for example a quarter of births are induced in the United States of America [2–4]). Induction is undertaken for a variety of reasons, frequently due to prolonged pregnancy [5] and pre-labour rupture of the membranes at term [6]. The evidence of the safety and effectiveness of IOL in improving outcomes has grown [7]. Evidence suggests that inducing women in additional risk groups would improve outcomes, and has driven a further increase in the induction rate, for example for women with hypertension [8], diabetes in pregnancy [9], and advanced maternal age [10, 11].

Maternity services in the UK are provided by the National Health Service, a universal health service free at the point of use. The majority of births occur in hospital, with antenatal and postnatal care provided in hospital and community clinics, and during home visits by midwives. Low risk care in the hospital and community is predominantly delivered by midwives, while care for women with more complex pregnancies is obstetrician-led. When a decision to induce labour is made, women attend the hospital for the procedure once a bed is available. Women wait in a ward or designated induction area, and a vaginal examination is undertaken to assess the cervix and whether artificial rupture of membranes (ARM) can be undertaken or whether intervention is necessary to prepare the cervix for labour. If further cervical ripening is necessary, usually vaginal prostaglandins are administered to ripen the cervix, or alternatively a balloon catheter or osmotic dilator may be inserted into the cervix. Subsequently, the woman is transferred to the Labour Ward (Delivery Suite) either because she has progressed to established labour, or for ARM if spontaneous labour has not occurred. If required, intravenous oxytocin may be given to stimulate uterine contractions.

Updated national guidance advising IOL at the earlier gestation of 41 weeks [12] rather than up to 42 weeks [5] increasing rates of IOL have occurred at the same time as substantial pressure on maternity service providers in the UK [13]. Maternity service leaders have expressed concerns that workforce shortages are impacting upon quality and safety of care [14, 15]. In addition, the Ockenden Review identified a number of instances of harm, including fetal death, following delays in transferring women undergoing induction to labour ward where the unit was busy [16]. Previous studies have explored the effectiveness of IOL in reducing clinical risk, and different strategies for IOL, along with staff and women’s perspectives of IOL, and recently a cross-sectional survey of UK maternity units in 2020 explored changes in IOL practice in response to the COVID-19 pandemic, focused on how the pandemic had changed cervical ripening practice, finding that a minority of maternity units had changed their methods and criteria, and that more women were returning home during cervical ripening [17]. Wider elements of local IOL policy and practice in UK maternity units are not known. The aim of this study was to explore key features of local IOL pathways and challenges in the UK National Health Service, in order to provide insight into current practice, variation between units, and opportunities to improve pathways and care.

## Methods

An online cross-sectional survey was developed by a multidisciplinary group consisting of researchers, senior midwives and consultant and trainee obstetricians. The survey was developed and administered involving members of the UK Audit and Research Collaborative in Obstetrics and Gynaecology (UKARCOG), a network of doctors in specialty obstetrics and gynaecology training, which conducts audit and research in UK NHS hospitals. UK maternity services sit within a range of administrative organisations: NHS Hospital Trusts (England and Wales), Health Boards (Scotland and Wales) or Health and Social Care Trusts (Northern Ireland). The Trusts and Boards can include one or more hospital sites providing maternity services, commonly referred to as ‘maternity units’. There is a UKARCOG trainee doctor working in most UK units, though the number of units without a representative is not known and fluctuates as doctors move between training placements. Previous UKARCOG projects have reported data from 148 and 76 NHS maternity units [18, 19].

All UK maternity units employing a UKARCOG doctor were eligible to take part, and all UKARCOG doctors were sent information to share with their employing unit regarding participation. A convenience sample of 71 units, each in a different NHS Trust or Board, agreed to take part, of which 54 completed the survey (response rate 76.1%). The convenience sample was by unit, rather than by doctor, with one response per unit.

Survey data were collected using the onlinesurveys.ac.uk platform [20]. The survey link was disseminated by the UKARCOG network, and completed by UKARCOG member in their employing hospital, with local agreement from the obstetrician in charge of Labour Ward/Delivery Suite. The survey included questions about maternity unit characteristics, IOL rates, local policies and processes, delays and incidents, with space for free text comments (survey questions provided in S1 File). The survey commenced in October 2021 and closed at the end of March 2022. UKARCOG assigned each unit a unique code which was included in the survey to facilitate sending of reminders. The research team did not know the identity of the participating units.

Data were downloaded into Microsoft Excel database by researchers at the University of Birmingham. For quantitative data descriptive statistics were computed. Frequencies and percentages were reported for categorical measures. Means and standard deviations or medians and interquartile ranges were reported for numeric measures. To explore for associations between categorical measures, Fisher’s exact tests were performed and p-values were reported. To explore for associations between numeric and categorical measures, ANOVA models were used and p-values were reported. The overall Induction rate from March 2020 to April 2021 was given as a percentage. Size of unit was categorised by the number of births in unit per annum (1,000–2,999; 3,000–4,999; 5,000–6,999 or 7,000+). Frequency of delays and causes of delays were categorised into the following groups: never; rarely; sometimes; often or always. Content analysis of free text was undertaken to produce a quantitative description of reasons for offering IOL. Researchers then identified which of the reasons aligned with national guidelines. The remaining free text data were reviewed and discussed by the research team, and did not warrant formal analysis, and therefore are not reported in this paper.

Ethical approval was secured from University of Birmingham Ethics Committee (ERN_21–0609) and the study was registered with each participating organisations’ service evaluation system. The UKARCOG doctor completing the survey on behalf of their organisation provided electronic confirmation that their obstetric lead doctor had consented to participation. No individual patient or staff data was collected therefore no informed consent was sought from an individual.

### Patient and public involvement

The project was discussed with the ARC West Midlands Maternity Theme Patient and Public Involvement and Engagement (PPIE) group during the study planning process, and they were supportive of the proposal. They were not involved in the development of the questionnaire as it was clinician-focused, which was designed with clinicians.

## Results

### Response rate and maternity unit characteristics

Fifty four responses were received (76.1% of units who initially agreed to take part). Responses were received from 35% (54/153) of UK NHS Trusts and Boards (though only one unit responded in each trust, see limitations section). Table 1 presents geographical location, size and level of care offered by participating units alongside all units in the UK, indicating representation from all types of unit.

**Table 1:** Comparison of location, size and neonatal care level* of participating maternity units with units across the UK.

	Type and size of unit	Participating NHS Trusts and Boards**	% n = 54	All UK NHS Trusts and Boards	% n = 153
Location	England	40	74	126	82
	Scotland	10	18.5	14	9
	Wales	3	5.5	7	4
	Northern Ireland	1	1.8	3	2
	Islands	0	0	3	2
Maternity unit size and neonatal level (five mutually exclusive, separate comparator groups developed by MBRRACE [21]	<2,000 births at 24 weeks or later, no level 3 NICU	4	7	20	13
	2,000–3,999 births at 24 weeks or later, no level 3 NICU	18	33	38	25
	4,000 or more births at 24 weeks or later, no level 3 NICU	12	22	42	28
	Level 3 NICU	9	16	28	18
	Level 3 NICU & neonatal surgery	11	20	25	16
	TOTAL	54	100	153	100

*As a proxy for level of clinical specialisation

**Note only one unit responded per Trust or Board, and some Trusts/Boards have more than one unit

**Fig 1:**
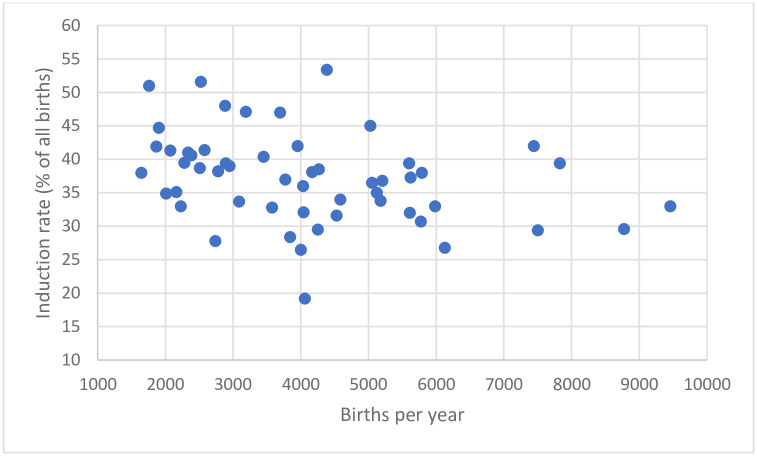
Induction rates according to maternity unit size (number of births per year).

IOL rates across the units varied substantially, with a median rate of 36.3% (IQR: 33.5% to 39.7%) and ranged from 19.2% to 53.4% (see Fig 1). There was no evidence of an association between the size of unit and induction rate (ANOVA, p = 0.100).

### Criteria for IOL

Most units reported that their organisation had agreed criteria for offer of IOL (39/54, 72%), presented in Table 2 which indicates which criteria appear in National Institute of Care and Excellence Guidelines (NICE) for IOL (versions published in both 2008 and 2021) [5, 12]. The most common criterion was prolonged pregnancy, with 33 of 39 responding units (85%) reporting this, and in a subsequent question all 54 units reported a specific gestation when IOL was offered for prolonged pregnancy. Other common criteria included maternal diabetes, and fetal growth restriction (FGR) (both 33/54, 85%). Where national guidelines recommended IOL for specific combinations of risk factors, e.g. where FGR *and* fetal compromise, responses to the survey often listed only one risk factor. Some respondents reported local IOL criteria which did not appear in national guidelines, such as assisted conception (n = 16), oligohydramnios / polyhydramnios (n = 14), social reasons (n = 9) or precipitate labour (where women have a history of rapid labour and IOL is offered to avoid birth unattended by health professionals) (n = 2).

**Table 2:** Local criteria for IOL in participating units (n = 39).

Induction of labour criteria in unit guidelines	Units reporting reason (n = 39)		Alignment with national clinical guidelines (with citation)			
	n =	%	NICE IOL guidelines (2008 & 2021)	In other NICE guidelines	In other guidelines	Not in guidelines
Prolonged pregnancy	33	85				
Pre-labour rupture of membranes (PROM) / pre-term PROM	29	74				
Fetal macrosomia / Large for gestational age	16	41				
Maternal request	10	26				
Previous caesarean section	6	15				
Breech / unstable lie	3	8				
Fetal growth restriction (FGR)	33	85			[22]	
Intra-uterine death (IUD) / previous IUD or poor outcome	21	54			[23]	
Fetal compromise: abnormal Doppler / absent end diastolic flow / meconium	7	18			[24]	
Diabetes	33	85		[25]		
Pregnancy Induced hypertension / pre-eclampsia / hypertension	28	72		[26]		
Multiple pregnancy	18	46		[27]		
Pre-existing maternal condition e.g. renal, cardiac, autoimmune disease	10	26		[28]		
Reduced fetal movements	29	74		[24]	[29]	
Obstetric cholestasis	27	69			[30]	
Maternal age >39 years old	24	62				
Assisted conception / risk factors for subfertility	16	41				
Oligohydramnios / polyhydramnios	14	36				
Antepartum haemorrhage	9	23				
Social risk factors / late booking	9	23				
Pelvic girdle pain	8	21				
Congenital anomaly / fetal abnormality	5	13				
Low Papp A	3	8				
Mental health concerns	3	8				
Precipitate labour	2	5				

### Gestation at IOL for prolonged pregnancy

NICE (2021) [12] recommends offering IOL between 41 and 42 weeks for women with uncomplicated pregnancies. All units responded to the question on gestation at IOL for prolonged pregnancy, and all followed guidance, though we note only 33 units reported induction for prolonged pregnancy in the previous guideline criteria question (see Table 2). The median gestation for IOL for prolonged pregnancy was 41^+5^ weeks (range of 41^+0^–42^+0^).

### Local approaches to clinical IOL decision-making

Participants were asked about the approach to making decisions to book women for IOL in their unit, including the use of multidisciplinary meetings, specific local requirements for the role and seniority of decision-makers, and decision-making where indications for induction were outside of agreed criteria (where criteria were available). Three units (6%) routinely held formal multidisciplinary meetings (involving at least one consultant obstetrician and midwife), some did so only if there were service pressures (n = 13, 24%), and most (n = 38, 70%) did not hold formal meetings. The majority (n = 42, 78%) of units required agreement from a consultant and/or senior registrar for IOL for any reason other than prolonged pregnancy. In 12 (22.2%) units, more junior registrars could agree IOL while in two, agreement was made by midwives for a defined group of women, such as those with pre-labour rupture of membranes.

Of the units with agreed local IOL criteria (39/54, 72%), most required one consultant to approve inductions for reasons not stipulated in agreed local policy (25/39, 64%), and 18% had no agreed process (7/39). Of the fifteen units without agreed IOL criteria, most (n = 12, 80%) stated that other national or local guidance was available (e.g. NICE), and two thirds (n = 10, 67%) reported variation in individual clinician practices for IOL decisions.

### The IOL booking process

Units used either an electronic booking system (28/54, 60%) or a paper diary (25/54, 46%) while one unit used both. Two units had no agreed threshold for the maximum number of inductions per day (see S1 Table), while for others the range was 2 to 15 each day, with larger units having higher limits.

### Where IOL takes place

Outpatient IOL (OP IOL, where women attend hospital for initial assessment and administration of cervical ripening agent, and then return home before reassessment in hospital) was available in 36/54 (67%) of units. The rate of OP IOL was recorded by only eight (22%) of these units (mean rate 30.7%, range of 2.7–81.0%). The most common reason for OP IOL was prolonged pregnancy (n = 27, 75%). Other reasons are provided as supplementary information (see S2 Table).

In two thirds of units (34/54, 63%) women who were suitable were offered transfer to a midwifery-led unit following IOL and labour starting. This was not offered in a fifth (11/54, 20%) of units (no response was provided by nine units).

40 (74%) units had a specific inpatient area where women started the IOL process. In free text responses, 18 (33%) reported that this location varied according to women’s risk status. Locations included antenatal ward beds (n = 23), a dedicated IOL ‘bay’ (n = 17), DS rooms (n = 7), and Day Assessment/Triage Unit (n = 6).

### Delays in the induction pathway

Admission for IOL was delayed due to a lack of available induction beds ‘often’ in 19% of units (n = 10) (see Table 3). There was no association between frequency of this delay in admission and size of unit (Fisher’s exact test, p = 0.227) or induction rate (ANOVA, p = 0.6214). While three units (6%) had an agreed definition for the threshold for delay in a bed being available to start the induction process in local guidelines, most did not. The definition of delay ranged from 2 to 24 hours. Most units (n = 40, 74%) had an agreed process in the guideline for monitoring women waiting for admission.

**Table 3:** Frequency of delays in IOL processes.

	Frequency of delays in IOL process n = 54 (%)				
	Never	Rarely	Sometimes	Often	Always
Delay in admission for IOL due to lack of beds (13 units reported ‘Not applicable’ and 1 did not respond)	1 (2)	10 (19)	19 (35)	10 (19)	0 (0)
Delay in moving women to DS for ARM or if membranes ruptured	3 (6)	2 (4)	15 (28)	30 (56)	4 (7)

Delays in women being transferred to DS once IOL was in progress (either for ARM or if membranes ruptured) were reported more frequently than delays in admission due to bed availability, with over half (n = 30, 56%) of units reporting this occurring ‘often’ and 4 (7%) ‘always’. 17 (31.5%) units had an agreed guideline definition of delay when women were clinically ready for transfer to DS. Of the 15 units who reported a specific defined time, there was a wide range (>2 hours to 5 days) with a mean of 14 hours 40 minutes. There was no association between delay in moving to Delivery Suite and the size of unit (Fisher’s exact test, p = 0.303) or induction rate (ANOVA, p = 0.1249).

The most frequent cause of delays reported by units was staffing levels (n = 41, 76% ‘always’ or ‘often’). Lack of physical space caused delays less frequently (n = 42, 78% ‘sometimes’ or ‘often’) while for units, neonatal capacity ‘never’ or ‘rarely’ caused delays in most units (n = 38, 70%) (see S3 Table). There was no evidence of an association between size of unit and the reason contributing to delays, for staffing levels (Fisher’s exact test, p = 0.304), lack of physical space (Fisher’s exact test, p = 0.123) or neonatal capacity (Fisher’s exact test p = 0.063). Other reasons for delays in 19 free text responses included workload/emergencies, increase in induction rate, unit acuity (the workload/complexity of cases), COVID-19, and prioritising of high-risk women.

### Incident reporting

In the past three months most units had triggered incident reports resulting from delays during induction (36, 67%) and 25 (46%) units reported formal complaints from women relating to IOL. Three (6%) units reported ‘episodes where there has been harm to mother or baby relating to delay in the induction process (including Serious Incident Reports and NHS England’s Healthcare Safety Investigation Branch reports)’. IOL had been identified as an area of concern by 24 (44%) units, 13 of which had placed it on the risk register, with 20 units (37%) reporting that the NHS Trust Board or NHS Scotland Board was aware that IOL was an area of concern. 33 (61%) units reported undertaking Quality Improvement projects in IOL.

## Discussion

### Principal findings and implications

Induction rates reported in our survey varied widely between units, including units of similar size. This variation was consistent with the latest National Maternity and Perinatal Audit which explored hospital records in England and Wales, though it reported an induction rate of 33.5% in England and Wales in 2019, lower than the median rate in our 2021 study (36.3%) [31]. There was variation in practice across all the units. Regarding the reason for induction, over a quarter of units did not have a single agreed list of reasons for IOL, and for those that did their reasons varied. Home cervical ripening was offered in two thirds of responding units, though most did not report the proportion of women receiving this option, and rates varied widely. A recent UK qualitative study of clinician perspectives of home cervical ripening suggests that while many units offer this option, rates are low [32], and a linked cross-sectional survey of women’s experiences also indicates that while some women welcome this option it may not be acceptable to all [33]. No delays in subsequent admission were reported specifically for women undergoing home ripening, though units were not asked about this directly. In view of the reported delays in securing DS beds for women already in the hospital, if increasing numbers of women are offered home cervical ripening it is important that beds are available in the hospital once they are in established labour.

Some criteria for IOL did not appear in the main NICE IOL guideline [12], and were instead located in other guidelines [12, 22–30, 34]. Guidance also deals with single indications, while increasing complexity and multimorbidity in pregnancy mean that women often have multiple risks and indications for IOL. This makes evidence-based decision-making complex, requiring awareness and integration of multiple NICE guidelines. Most local criteria reported in our survey were consistent with NICE, but some, for example, assisted conception, were not. Our findings also suggest that some IOL indications which appear in NICE are missing in local guidance. The local variation may drive inconsistency in practice between maternity units, and hamper learning and consistent decision-making for staff who move around units, such as doctors in training.

It is important to acknowledge that ‘guidance’ is not mandatory, and that some variation in practice will always be required to personalise care, where an NHS trust does not provide a clinical service (e.g. OP IOL), or where circumstances are complex and nuanced, but our findings suggest that more standardisation of IOL guidelines is needed. The variation we have identified in UK maternity units is consistent with the findings of a systematic review of international IOL guidelines by Coates et al [35], which identified substantial variation in recommendations and highlighted the uncertainty and emerging evidence base in many areas.

There was variation in clinician involvement in the decision-making for induction with a minority of units reporting use of multidisciplinary teams or two-consultant agreement for IOL decisions. The majority of units reported a single consultant agreement while some had no agreed process, suggesting that decisions are predominantly based on individual clinician judgement. This indicates that there are opportunities for strengthening collaborative decision-making. Most units offered outpatient induction, and for low-risk women offered transfer to a midwifery led setting once labour had started, but around a third of units did not do so, suggesting that there are opportunities to widen access for women. However, it is also important to note that while services reported outpatient induction and midwifery led settings, we do not have evidence on how often these options are actually offered in practice. Ongoing work is exploring the safety, costs and acceptability of outpatient induction in the NHS [36], and expansion of this approach may enable units to manage inpatient capacity and IOL delays more effectively. Almost half of units reported using a paper diary to book and manage IOL capacity, and the current national investment in electronic data systems in UK maternity services can offer a solution to improving digital management of capacity to visualise and prioritise women waiting [37]. A minority of units did not have a maximum induction threshold, which may indicate a safety issue, and may lead to delays in the IOL process when many women are booked for an IOL in a short time frame.

Delays due to a lack of available beds were reported by over half of units, with delays in DS beds for women whose membranes had ruptured or required ARM the most common challenge, and workforce issues were the most frequently reported cause of delays in the process for women in our survey. The wide variation in the local definition of ‘delay’ suggests an urgent need to standardise definitions, at a time when a review into avoidable deaths in NHS maternity care highlighted that long waits may be normalised by clinicians, and recommended that “*Trusts [hospitals] need a mechanism to clearly describe safe pathways for IOL if delays occur due to high activity or short staffing*” [16]. Staffing problems present an acute challenge to providing safe and personalised maternity care [16, 38, 39] and have been implicated in incidences of harm for women and babies undergoing IOL [16, 38, 39]. Within complex health systems there is always a risk of unintended consequences when changes are introduced [40]. Changes to national clinical guidelines which are intended to improve outcomes have increased demand for IOL services in the UK, without concurrent increases in capacity, and the resulting service pressures may mean the expected outcomes from increasing IOL may not be realised. As Weeks and Alfirevic have highlighted, it cannot be assumed that the effectiveness of IOL demonstrated in clinical trials will be observed in all contexts, particularly in low resource settings [41], but as workforce pressures mount globally this is also an important consideration for high income countries. This bottleneck in the pathway presents opportunities for focused quality improvement [42] and warrants further exploration.

Most units reported incidents and almost half formal complaints, though a minority reported harm. It is possible that the incident and harm rates are higher, as recent reports have indicated that reporting statistics do not always accurately reflect event rates [43, 44]. There is potential for psychological harm to women as a result of delays in IOL, including distress caused by fears about the impact on their baby [24], which may not be recorded as a formal complaint or incident. Given the low frequency of perinatal death induction is designed to avoid, it is also possible that the harm occurring during induction delays is as great as the potentially avoidable fetal deaths induction is targeted at reducing. It is clear that many units (over half of our sample) are undertaking quality improvement projects to improve IOL care, though we did not gather detailed information, and they are likely to vary substantially in extent and quality [45]. Overall, the variation in practice and policy highlighted by this survey indicates that there is a need for more standardisation. This together with identified staffing issues suggest safety concerns for IOL care in many maternity units. Given the uncertainty as to whether within NHS settings mode of birth is unaffected by IOL, as suggested in RCTs, it is vital that national and local outcomes for IOL are included in information for women. This is essential in order that women can make informed choices between any potential risks associated with pregnancy progression and the process and birth outcomes associated with ‘real life’ IOL in NHS settings.

### Strengths and limitations

This is the first study to provide a UK-wide report of concerning variation in local guidelines, practice and delays in induction of labour in the NHS, and the findings add to the limited evidence recently published. Responses represent more than a third of NHS Trusts and Boards across all countries of the UK, and are broadly representative of all units in terms of size and neonatal specialist service provision. However, it is important to note that we only received one response from any single Trust/Board, therefore some Trusts/Boards are only partially represented. We explored the key characteristics of units (country, number of births, neonatal care provision), though there may be other differences between our sample and the wider NHS which impact on IOL practice which are not known, and the anonymous nature of the survey prevents further interrogation of differences. Findings complement existing national audit data which reports induction rates only, for 89% of births in England and Wales. The anonymous and independent data collection was designed to encourage openness in sharing local challenges. However, the anonymity limited the available information regarding case mix of the populations served, and clinical complexity may impact on induction rates and care pathways. We sought expert clinical input into survey design to maximise the relevance and accuracy of data collected, though there may have been some reporting errors and/or bias. The updated 2021 NICE IOL guidelines [12] were published during the course of data collection, and some units may have since updated their own local guidance.

## Conclusion

Induction of labour is a common and important intervention to safely manage risk for women and babies in maternity care. A key message from this study is that there appears to be substantial variation in local policy and practice with regard to IOL across UK maternity units, which may be addressed through development and implementation of standardised guidance and pathways across maternity systems, including rigorous evaluation. There are also challenges and delays in providing care. Evidence has widened the indications for IOL, and rates continue to increase, at the same time as severe workforce pressures within maternity services. Our study highlights a need to support local maternity units to provide a safe, effective and woman-centred IOL pathway.

## Supporting information

S1 File Induction of labour survey questions. (DOCX)

S1 Table Maximum number of inductions able to be booked per day per size of unit. (DOCX)

S2 Table Criteria for outpatient IOL across responding units. (DOCX)

S3 Table Reasons contributing towards delays in the induction process. (DOCX)

## Abbreviations

IOL: Induction of labour
NHS: National Health Service
PPIE: Patient and Public Involvement and Engagement
