# Supplementary material for: Induction of labour care in the UK: A cross-sectional survey of maternity units
Source: PLoS One. 2024 Feb 28;19(2):e0297857. doi: 10.1371/journal.pone.0297857 (PMC10901341; doi:10.1371/journal.pone.0297857)
Supplement: S1 File — (DOCX) [file pone.0297857.s001.docx]

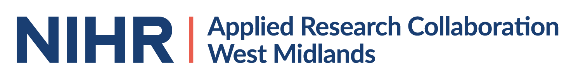

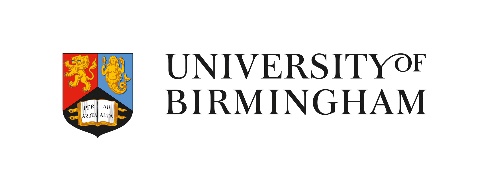


**Supplementary Information:**

S1 – Induction of labour survey questions

**Survey of UKAROG Maternity units regarding the process of Induction of Labour**

This survey is part of work being undertaken by the Maternity Theme of the NIHR Applied Research Collaboration in West Midlands (ARC WM) based at the University of Birmingham led by Professor Sara Kenyon to explore the nature and extent of any logistical problems present in the process of induction of labour in clinical practice.

Induction of labour is defined as the process by which labour is started prior to its spontaneous onset by progressive cervical effacement and dilatation and/or artificial stimulation of uterine contractions, leading to active labour and birth.

In order to complete the questionnaire the best person to contact locally is the obstetrician who leads Labour Ward/Delivery Suite- either they or the Midwifery Matron/Operational Midwife lead will be able to direct you to the person who will be able to provide the data we are requesting.

**Individual Trusts or individuals will not be identifiable in any reports or publications and your responses are confidential.**

**Please confirm that this project has been registered on the Trusts internal systems for service evaluations and that consent has been obtained from the Obstetric lead for Delivery Suite for the data to be submitted**

Confirmation box

1. **ABOUT YOUR UNIT**
2. **Unit code ______________________** (given by UKARCOG)

**Which definition best describes your unit? (these are definitions used by MBRRACE UK, select all that apply)**

Level 3 NICU and neonatal surgery Level 3 NICU

4,000 or more births per annum at 24 weeks or later 2,000-3,999 births per annum at 24 weeks or later

Under 2,000 births per annum at 24 weeks or later

1. **How many births did you have between March 2020-April 2021 (financial year)? _________**
2. **What is your overall induction rate in March 2020- April 2021 (%)? ______ %**
3. **Is local induction data available between March 2020- April 21 for nulliparous and multiparous women?**

**Yes No**

Nulliparous women ______%

Multiparous women ______%

1. **INDICATIONS AND PROCESSES FOR OFFERING INDUCTION**
2. **Is there a single agreed list of reasons for offering induction to women in your unit? (this may be on your electronic patient record)**

No Yes (if so, please can you list in the comment box )

Comments

1. **If there is an agreed list, is there a process for agreeing the reason for induction *outside* this?**

No Consultant only agreement Two consultants in agreement

Other (please list here)

1. **If there is no agreed list, why is this?**

Variation between individual clinician practices regarding indications for offering induction

Don’t know

Other (please describe here)

1. **If there is no agreed list, is there any other guidance about reasons for induction?**

Yes No

Comments

1. **Is there a formal MDT (Consultant Obstetrician and Midwife) that reviews all the cases for induction before they are booked?**

Yes No Only if there is a capacity issue identified

Comments

**Aside from a routine post-dates induction, does a consultant +/- senior registrar have to agree to the induction?**

Yes No

Please provide details here

1. **Is outpatient induction offered to women? (defined as home cervical ripening, whereby women attend hospital for initial assessment and administration of cervical ripening agent; and then return home before reassessment in hospital)**

Yes No (if no, please go to Question 15)

Comments

1. **Can you tell us your eligibility criteria for outpatient induction?**
2. **How many women started the induction process as an outpatient between March 2020- April 2021?** ______%
3. **If low risk women go into labour during the induction process (prior to requiring syntocinon) can they go the Midwife Led Unit/ Birth Centre (MLU)? (For all women, prostaglandin and mechanical induction.)**

Yes No Not applicable (no midwife led unit)

1. **Is there an identified area where the induction is undertaken for inpatients? E.g. induction bay before move to delivery suite.**

Yes No

If yes, please provide details here

1. **Are there different areas for low risk and high risk inpatient induction?**

Yes No

1. **At what gestation do you routinely induce post-dates,(weeks and days e.g. 35+5)?**
2. **What booking system do you use for women whose induction is agreed?**

Electronic booking system Paper diary Both electronic booking system and paper diary None

1. **Is there a planned limit of how many inductions can be booked in a day? NB we appreciate that this may be exceeded due to clinical requirement**

Yes No

If yes, can you tell us what the limit is? ___________

1. **DELAYS IN THE INDUCTION OF LABOUR PROCESS**

Confirmation of a bed on the day of induction - please answer either question A or B (whichever is applicable)

1. **A. If women have to phone the maternity unit for a bed on the day the induction is booked, are there delays due to beds not being available (e.g. does the woman have to wait for a later date / come in at an unplanned time)?**

N/A Never Rarely Sometimes Often Always

1. **B. Do you phone the women to let them know there is a delay in a bed being available?**

N/A Never Rarely Sometimes Often Always

1. **Is there an agreed definition for the delay in a bed being available to start the induction process in your local guideline?**

Yes No

If yes, how long is this?____________________

1. **Does the guideline include a process for managing women who are at home if their induction is delayed before treatment is started ?**

Yes No

Comments

1. **Once the induction process is underway and the woman is clinically ready for ARM or if membranes ruptured are there delays in a room being available on Delivery Suite?**

Never Rarely Sometimes Often Always

1. **Is there an agreed definition in your local guideline of this delay when women are clinically ready for transfer to Delivery suite?**

Yes No

If yes, how long is this?____________________

1. **REASONS FOR DELAYS IN THE INDUCTION PROCESS**
2. **Are staffing levels resulting in delays at any point in the process for women undergoing induction?**

Never Rarely Sometimes Often Always

Comments

1. **Is lack of physical space resulting in delays at any point in the process for women undergoing induction?**

Never Rarely Sometimes Often Always

Comments

1. **Is neonatal capacity resulting in delays at any point in the process for women undergoing induction?**

Never Rarely Sometimes Often Always

Comments

1. **Are there any other reasons for delays at any point in the process for women undergoing induction?**

No Yes

If yes, please provide details here

1. **INCIDENT REPORTING**

*You may find it helpful to sit down with your Labour Ward Lead to discuss data in this section as they will know where to find this information.*

1. **Have you had Incident Reports (e.g. Datix) regarding delays in the induction process in the last three months?**

Yes No

If yes, how many (if available)___________

1. **Has the unit received any formal complaints from women relating to induction of labour in the last three months?**

Yes No

If yes, how many (if available)___________

1. **Have there been any episodes where there has been harm to mother or baby relating to delay in the induction process (including Serious Incident Reports and HSIB reports) in the last three months?**

Yes No

If yes, how many (if available)___________

1. **Is induction of labour identified as an area for concern by the Directorate in your unit?**

Yes No

1. **If you answered yes to Question 35:**

Is it on the risk register? Yes No

Is the Board aware? Yes No

1. **IMPROVING INDUCTION PRACTICE**
2. **Has the Trust/Board undertaken any local QI projects in induction?**

Yes No

1. **Are there any examples of good practice or innovation in induction of labour that you are happy to share?**

Comments

1. **Is there anything else you would like to tell us that may be helpful for this project?**

Comments

**Once this survey is submitted it will not be possible to withdraw this information**

Thank you for taking the time to complete this survey
