# Supplementary material for: Induction of labour care in the UK: A cross-sectional survey of maternity units
Source: PLoS One. 2024 Feb 28;19(2):e0297857. doi: 10.1371/journal.pone.0297857 (PMC10901341; doi:10.1371/journal.pone.0297857)
Supplement: S1 Table — (DOCX) [file pone.0297857.s002.docx]

S2 - Table 1: Maximum number of inductions able to be booked per day per size of unit

| Annual number of births | Total number of units | Maximum number of inductions per day  Number of units (%) | | | | | | |
| --- | --- | --- | --- | --- | --- | --- | --- | --- |
|  |  | <4 (%) | 4 to 6 (%) | 7 to 9 (%) | 9 to 12 (%) | 13 to 15 (%) | No limit (%) | Not documented (%) |
| 1000-2999 | 19 | 11 (57.9) | 8 (42.1) | 0 (0.0) | 0 (0.0) | 0 (0.0) | 0 (0.0) | 0 (0.0) |
| 3000-4999 | 18 | 0 (0.0) | 15 (83.3) | 2 (11.1) | 0 (0.0) | 0 (0.0) | 0 (0.0) | 1 (5.6) |
| 5000-6999 | 12 | 0 (0.0) | 10 (83.3) | 1 (8.3) | 0 (0.0) | 0 (0.0) | 1 (8.3) | 0 (0.0) |
| 7000+ | 5 | 0 (0.0) | 0 (0.0) | 2 (40.0) | 1 (20.0) | 1 (20.0) | 1 (20.0) | 0 (0.0) |
| **Total** | 54 | 11 (20.4) | 33 (61.1) | 5 (11.1) | 1 (1.9) | 1 (1.9) | 2 (3.7) | 1 (1.9) |
