# Supplementary material for: Induction of labour care in the UK: A cross-sectional survey of maternity units
Source: PLoS One. 2024 Feb 28;19(2):e0297857. doi: 10.1371/journal.pone.0297857 (PMC10901341; doi:10.1371/journal.pone.0297857)
Supplement: S2 Table — (DOCX) [file pone.0297857.s003.docx]

S3 - Table 2: Criteria for outpatient IOL across responding units

|  | Number n=36 | **%** n=36 |
| --- | --- | --- |
| Postdates | 27 | 75.0 |
| Low risk | 24 | 66.7 |
| Private transport &/ or lives < 30 mins | 17 | 47.2 |
| Age <40 yrs | 16 | 44.4 |
| Parity <3 | 15 | 41.7 |
| GDM - diet controlled | 14 | 38.9 |
| Landline / mobile | 14 | 38.9 |
| Speaks English / no communication issues | 13 | 36.1 |
| Maternal request / social reasons | 11 | 30.6 |
| Large for gestational age baby | 11 | 30.6 |
| Normal cardiotocography | 11 | 30.6 |
| Symphysis pubis diastasis | 10 | 27.8 |
| Cephalic presentation / engaged in pelvis | 10 | 27.8 |
| Normal USS / not polyhydramnios | 9 | 25.0 |
| BMI normal or <40 | 8 | 22.2 |
| Singleton | 8 | 22.2 |
| Someone at home to be with woman while waiting to return to unit | 7 | 19.4 |
| Bishops score specification | 7 | 19.4 |
| Obstetric cholestasis | 6 | 16.7 |
| No medical conditions | 6 | 16.7 |
| No uterine surgery / previous caesarean section | 5 | 13.9 |
| Intact membranes | 5 | 13.9 |
| Primiparous only | 5 | 13.9 |
| No obstetric issues eg. antepartum haemorrhage, reduced fetal movements | 5 | 13.9 |
| No history of precipitate labour | 4 | 11.1 |
| No safeguarding issues | 4 | 11.1 |
| Fertility treatment/IVF | 4 | 11.1 |
| >37/40 gestation | 4 | 11.1 |
| Well controlled essential hypertension | 3 | 8.3 |
| Not inpatient | 3 | 8.3 |
