# Supplementary material for: Induction of labour care in the UK: A cross-sectional survey of maternity units
Source: PLoS One. 2024 Feb 28;19(2):e0297857. doi: 10.1371/journal.pone.0297857 (PMC10901341; doi:10.1371/journal.pone.0297857)
Supplement: S3 Table — (DOCX) [file pone.0297857.s004.docx]

**Supplementary Information:** Induction of labour care in the UK: a cross-sectional survey of maternity units

S4 - Table 3: Reasons contributing towards delays in the induction process

|  | Total number of units | How often reason contributes towards delays in induction process  Number of units (%) | | | | |
| --- | --- | --- | --- | --- | --- | --- |
|  |  | Never | Rarely | Sometimes | Often | Always |
| **Are staffing levels resulting in delays at any point in the process for women undergoing induction?** | | | | | | |
| 1000-2999 | 19 | 0 (0.0) | 0 (0.0) | 5 (26.3) | 14 (73.7) | 0 (0.0) |
| 3000-4999 | 18 | 0 (0.0) | 0 (0.0) | 5 (27.8) | 10 (55.6) | 3 (16.7) |
| 5000-6999 | 12 | 1 (8.3) | 0 (0.0) | 2 (16.7) | 7 (58.3) | 2 (16.7)_ |
| 7000+ | 5 | 0 (0.0) | 0 (0.0) | 0 (0.0) | 5 (100.0) | 0 (0.0) |
| Total | 54 | 1 (1.9) | 0 (0.0) | 12 (22.2) | 36 (66.7) | 5 (9.3) |
| **Is lack of physical space resulting in delays at any point in the process for women undergoing induction?** | | | | | | |
| 1000-2999 | 19 | 1 (5.3) | 7 (36.8) | 6 (31.6) | 5 (26.3) | 0 (0.0) |
| 3000-4999 | 18 | 0 (0.0) | 2 (11.1) | 9 (50.0) | 7 (38.9) | 0 (0.0) |
| 5000-6999 | 12 | 1 (8.3) | 0 (0.0) | 6 (50.0) | 5 (41.7) | 0 (0.0) |
| 7000+ | 5 | 0 (0.0) | 1 (20.0) | 4 (80.0) | 0 (0.0) | 0 (0.0) |
| Total | 54 | 2 (3.7) | 10 (18.5) | 25 (46.3) | 17 (31.5) | 0 (0.0) |
| **Is neonatal capacity resulting in delays at any point in the process for women undergoing induction?** | | | | | | |
| 1000-2999 | 19 | 4 (21.1) | 13 (68.4) | 2 (10.5) | 0 (0.0) | 0 (0.0) |
| 3000-4999 | 18 | 1 (5.6) | 11 (61.1) | 6 (33.3) | 0 (0.0) | 0 (0.0) |
| 5000-6999 | 12 | 1 (8.3) | 6 (50.0) | 2 (33.3) | 3 (25.0) | 0 (0.0) |
| 7000+ | 5 | 0 (0.0) | 2 (40.0) | 3 (60.0) | 0 (0.0) | 0 (0.0) |
| Total | 54 | 6 (11.1) | 32 (59.3) | 13 (24.1) | 3 (5.6) | 0 (0.0) |
